# Effects of High Hydrostatic Pressure on the Conformational Structure and Gel Properties of Myofibrillar Protein and Meat Quality: A Review

**DOI:** 10.3390/foods10081872

**Published:** 2021-08-12

**Authors:** Huipeng Liu, Yiyuan Xu, Shuyu Zu, Xuee Wu, Aimin Shi, Jinchuang Zhang, Qiang Wang, Ning He

**Affiliations:** 1 Department of Chemical and Biochemical Engineering, College of Chemistry and Chemical Engineering, Xiamen University, Xiamen 361005, China; m13526718841@163.com (H.L.); xuyiyuan@stu.xmu.edu.cn (Y.X.); zushuyu@126.com (S.Z.); xewu@xmu.edu.cn (X.W.); 2 Institute of Food Science and Technology, Chinese Academy of Agricultural Sciences, Yuanmingyuan West Road, Beijing 100193, China; shiaimin@caas.cn (A.S.); zhangjinchuang1002@163.com (J.Z.)

**Keywords:** myofibrillar protein, high hydrostatic pressure, gel properties, meat quality

## Abstract

In meat processing, changes in the myofibrillar protein (MP) structure can affect the quality of meat products. High hydrostatic pressure (HHP) has been widely utilized to change the conformational structure (secondary, tertiary and quaternary structure) of MP so as to improve the quality of meat products. However, a systematic summary of the relationship between the conformational structure (secondary and tertiary structure) changes in MP, gel properties and product quality under HHP is lacking. Hence, this review provides a comprehensive summary of the changes in the conformational structure and gel properties of MP under HHP and discusses the mechanism based on previous studies and recent progress. The relationship between the spatial structure of MP and meat texture under HHP is also explored. Finally, we discuss considerations regarding ways to make HHP an effective strategy in future meat manufacturing.

## 1. Introduction

Protein is one of the most important elements of nutrition for humans, providing energy and essential amino acids. Meat is the most valuable livestock product and, therefore, the preferred protein source of many people [1]. In addition, meat can be part of a balanced diet, providing valuable nutrients that are beneficial to health and essential for human growth and development [2,3]. Consumers seek meat products that are of a high quality, are safe to eat and are fresh. Safety and natural flavors with no additives (i.e., preservatives and moisturizers) are their primary requirements [4]. Based on this demand, a new processing technology was developed in the recent years known as high hydrostatic pressure (HHP), which shows the possibility for achieving better processing and preservation of meat, poultry and seafood [5].

HHP treatment is a non-thermal processing method that has been widely used in the fields of seafood, fruit, vegetable and meat products [5]. HHP is a completely physical process in which food is subject to uniform pressure treatment from all directions.

Treatment with HHP is not limited by the shape or size of the material, and the original shape of the solid food can be maintained [6]. Meat is mainly composed of water, protein and lipids [7]. The relatively high protein content (i.e., 16–40%) in meat plays an important role in food flavor, nutrition, and health [8,9]. Compared to the traditional heating process, HHP technology only affects the non-covalent structure in foods [10,11]. HHP can preserve freshness and nutritional values of foods by preserving vitamins and free amino acids and reducing losses of desirable compounds [12,13]. Therefore, the effect of HHP on protein in meat products is central to the importance of HHP technology in the manufacture of meat products.

Muscle protein mainly consists of myofibril protein, comprising two-thirds of the total proteins in lean muscle. In meat product processing, changes in MP will alter the quality of meat, including its appearance, hardness, springiness, chewiness, juiciness, taste and other texture characteristics [14,15,16]. Therefore, the effect of HHP on the structure of MP has been studied for many years. In this paper, research on the changes in MP under HHP in recent years is summarized. The relationship between MP structure and meat quality is discussed. The challenges and prospects for future applications of HHP in meat industry are also outlined.

### 1.1. Myofibrillar Protein (MP)

Myofibrillar protein, a salt-soluble protein, accounts for approximately 55% of muscle protein content and mainly includes tropomyosin, myosin, myogenic protein, and actin [17]. Myosin and actin are the most important functional and structural MP components that contribute to meat texture [18]. Myosin is a macromolecular protein with a shape similar to that of a bean sprout, and with a molecular weight of approximately 520 kDa, consists of two heavy chains and four light chains [17]. The myosin heavy chain forms the stem of the bean sprout, and the light chain forms the petals of the bean sprout [18]. In addition, the ATPase activity of myosin is correlated with muscle shortening speed [19]. Actin is an important component of thin filaments in MP, with a molecular weight of about 42 kDa [20], it exists in two forms: free monomer G-actin (spherical) and linear polymer F-actin (filamentous) [21]. Muscle contraction is actually the interaction between myosin and actin, in that actin provides the molecular motor, myosin hydrolyses ATP, to release energy, while filamentous actin acts as the track for myosin movement [18].

### 1.2. Changes in MP Structure and Gel Properties under HHP

#### 1.2.1. Effect of HHP on MP Structure

HHP can dissociate the non-covalent, ionic, hydrophobic, and hydrogen bonds to change the secondary, tertiary, and quaternary structure of MP [22]. Changes in pH, ionic strength, pressure and temperature can cause the denaturation, aggregation, or gelation of proteins [23]. The pressure level induces either local or global changes in protein, and eventually leads to denaturation by altering the delicate equilibrium of the interactions that stabilize the folded conformation of native proteins [24,25]. For example, the quaternary structure dissociates when treated at moderate pressures (100–200 MPa), the tertiary structure is significantly affected at a pressure level above 200 MPa, and secondary structure changes take place at higher pressures (300–700 MPa) [26]. According to Le Chatelier’s principle, pressure induces a shift of the equilibrium toward the state with the smallest volume [25]. The current study suggests that the main reason for the volume differences between folded and unfolded proteins is the solvent excluded void volumes that results upon unfolding [25]. Thus, the effect of pressure on any given protein varies depending on the specificity of its folding state (i.e., its internal packing density and pattern). Because hole volumes are not uniformly distributed in the folded polypeptide, homologous proteins with the same size and structure may have different volume properties and, thus different sensitivity to pressure perturbations [25]. HHP induces variable alterations on protein conformational structures depending on the applied pressure level [26]. Table 1 provides recent reports on the changes in MP structures with HHP treatment.

#### 1.2.2. Secondary Structure

By using available computational methods, detailed pressure-induced changes in protein secondary structure from characteristic shifts in the band frequencies that are recorded via various techniques, such as Fourier-transform infrared spectroscopy (FTIR), Raman spectra and circular dichroism (CD) [27]. The secondary structure of protein includes α-helix, the β-sheet, β-turn, random coil [28]. The α-helix is stabilized by intramolecular hydrogen bonds between the carbonyl oxygen and amino hydrogen of the polypeptide chain and is buried in the interior site of the protein [29]. The β-sheet is organized through intermolecular hydrogen bonds. The β-turn structure is usually distributed on the surface of a protein molecule, where the change in direction of the polypeptide chain encounters less resistance, and protein stability is maintained through the relationship between the amino acids and the electrostatic gravitational forces. A random coil can originate from the unfolding of any α-helix, β-sheet and β-turn structures and contributes to protein flexibility [30,31]. 

Most studies have shown that 100 MPa pressure can change the secondary structure of MP in meat products, with specific details according to various levels of pressure outlined in Table 1. However, some experiments have also demonstrated that the secondary structures of MP and tropomyosin are not significantly affected when the pressure is 100 MPa [32]. Schiaffino et al. pointed out that MPs from different sources have slight diversities within their amino acid sequences, leading to different structures and differences in the contractile and biochemical properties of the muscle, which may affect their response to pressure [33]. 

In a previous report, the α-helix structure of MP was significantly reduced by HHP, with corresponding increases in the contents of β-turn and random coil [26]. Some studies have suggested that HHP induces water infiltration into protein cavities, strengthening the hydrogen bonds between the protein and water molecules, wakening the intramolecular hydrogen bonds, resulting in changes to protein configuration [34,35]. With an increasing in pressure level, a gradual increase in β-sheets content is normally observed, but in the case of tropomyosin, the β-sheets content decreased at lower pressures and increased at higher pressures [36]. Li et al. attributed this to the fact that, at a relatively lower pressure, intermolecular hydrogen bonds could be broken, rather than intramolecular hydrogen bonds [36]. With an increase in pressure, the intramolecular hydrogen bonds would be destroyed, leading to the transformation of α-helix to β-sheet, β-turn, and random coil, thus resulting in an increase of β-sheet content. It has also been found that abalone proteins are predominantly of β-sheet structure [28]. HHP could disrupt the intermolecular β-sheet structure in abalone proteins, which was compensated for by forming new intramolecular β-sheet interactions [28]. Chen et al. found that the β-turn was reduced with the treatment of pressure [37]. In general, highly ordered structures (α-helix, β-sheet and β-turn) unfold under HHP, resulting in an increase in random coil structure [26]. Decreased α-helix is more common under HHP, but the pressure threshold for the reduction is closely related to the environment and the protein source [26]. HHP changes the MP secondary structure because the high pressure breaks its intermolecular hydrogen bonds structure and thus weakens the intermolecular interactions, while intermolecular and intramolecular interactions can be reformed in various combinations, resulting in the reduction, increase or retention of secondary structure conformation.

#### 1.2.3. Tertiary and Quaternary Structure

Changes in surface hydrophobicity (H_0_) and the contents of the free sulfhydryl group (−SH) reflect changes in the tertiary structure of proteins and are closely related to their functional properties [38]. In an aqueous environment, nonpolar solutes repel water, which is one of the properties of hydrophobic groups [39]. The hydrophobic interaction between the side chains of nonpolar amino acid residues is one of the main forces that maintains the tertiary structure of proteins, and it plays an important role in protein functions [40]. The ANS fluorescence probe method is a method to evaluate protein surface hydrophobicity [41]. According to Table 1, the surface hydrophobicity and reactive sulfhydryl groups (−SH) of MP increase under HHP. A possible reason for this is that the tertiary structure of MP is destroyed, resulting in the internal exposure of hydrophobic groups under HHP [42]. Since the disulfide bond destruction requires 213.1 kJ/mol of energy, and the 10,000 MPa pressure treatment can only provide 8.37 kJ/mol of energy, the disulfide bond will not be destroyed by HHP treatment [43,44]. Thus, the increasing in the content of reactive −SH may be due to the unfolding the protein structure and the exposure of internal sulfhydryl groups [26,44]. In our work [9], it was found that when the pressure exceeded 400 MPa, the surface hydrophobicity content of MP began to decrease, possibly due to the aggregation of proteins that are covered by random amino acid residues. Bolumar et al. decribed the mechanism of MP aggregation in detail as follows: (a) Dissociation of the thin and thick filaments caused MP solubilization increased; (b) rupturing of non-covalent interactions inside the molecules results in protein denaturation; and (c) formation of new intra- and/or intermolecular bonds [18]. 

The quaternary structure of proteins is mainly stabilized by pressure-sensitive hydrophobic forces, which disassemble oligomeric proteins of pressures at 100–200 MPa, accompanied by a reduction in volume [45]. Thus, HHP treatment causes protein depolymerization and disruption of the hydrophobic bonds between the linked polypeptide chains, leading to the dissociation of numerous polypeptide chains [45]. Under HHP treatment, water molecules enter the binding region of each subunit, affecting non-covalent bonds (especially hydrophobic interactions) and destabilizing the connections between subunits, resulting in depolymerization, and affecting the quaternary structure of the proteins [26]. The dissociation of oligomeric proteins can be promoted by pressure treatment below 150 MPa. Pressure above 150 MPa can cause the protein to dissociate and the oligomeric subunits to recombine after separation [46].

**Figure 1:**
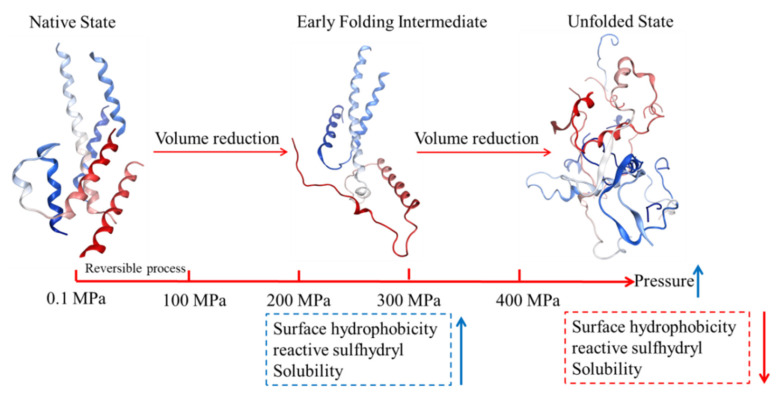
Schematic diagram of the mechanism of pressure-induced protein denaturation.

Figure 1 shows the mechanism of MP structural changes under HHP. HHP provokes changes in molecular interactions (hydrogen bonds, hydrophobic interactions, and electrostatic bonds). This leads to significant conformational changes of in the myofibrillar protein structure (secondary, tertiary and quaternary) based on the volume reduction between folded and unfolded MP, as well as the dissociation of MP. The α-helix undergoes pressure-driven transformation into a random coil and β-turn, and higher pressures induced greater denaturation and unfolding of MP. The increase in H_0_ is attributed to the HHP-induced unfolding of MP. Mild and moderate pressure during HHP treatment exposure hydrophobic and sulfhydryl groups, which is beneficial for protein–protein interactions. Excessive pressure (above 400 MPa) causes MP aggregation due to the intermolecular interactions that dominate [40,47,48]. Overall, the reduction of free volume in the MP network under HHP contributes to the conformational rearrangement of secondary, tertiary and quaternary structures.

### 1.3. Effect of HHP on Myofibrillar Protein Gel 

The gel properties of MP determine the texture, adhesion and water retention of meat products. The formation of protein gels usually involves denaturation and aggregation, that is, the formation of a network structure, through physical and chemical reactions, that is capable of retaining water, fat, or other components [14]. In order to improve the quality of meat products and their gelatinous forms, polysaccharides are normally added [56]. Furthermore, new processing technologies, such as HHP, are also applied [16]. Table 2 summarizes recent studies on the mechanism of HHP-induced myofibrillar protein gelation.

As discussed in Section 1.2, HHP treatment induces MP unfolding. Moderate high pressure and a low concentration of CaCl_2_ can change the physical properties of meat products, mainly because, at pressures under 200 MPa, a large amount of myosin heavy chains and actin are dissolved, protein aggregation ability is reduced, and the tyrosine and tryptophan residues are exposed due to the unfolding of the tertiary structure [57]. Hsu et al. measured the aggregation and viscoelasticity of myosin in tilapia (*Oreochromis niloticus*) at 0° [65]. At 500 atmospheres, myosin unfolds; at 1000 atmospheres, they cling together, while at pressures higher than 1500 atmospheres, myosin forms aggregates and gels [65]. Chen et al. investigated the relationship between the gel properties of surimi and the structure of MP under high pressure (300~450MPa) [37]. The results showed that the secondary structure of MP in the surimi gels was significantly correlated with the whiteness, strength and structural characteristics of the gels. In general, pressure induces the unfolding of the protein, exposing more hydrophobic amino acids, enhancing the hydrophobic interaction and forming a three-dimensional network structure [26]. The enhanced solubility of the MP helped to improve its gel properties [66]. Zhang found that the content of disulfide bonds in MP gels was positively correlated with the treatment pressure [44]. Some studies showed that the pressure-induced protein gelation is also based on the formation of disulfide bonds [58,61]. However, Wang et al. reported that high pressure induced the hydrophobic rearrangement of MP and the cross-linking of disulfide bonds in the myosin S-1 subsegment [57]. This resulted in the formation of large protein aggregates and a decrease in MP solubility, which indicated a weaker gel and a lower water-holding capacity. For cod and turkey muscle, the gelation mechanism is similar to that of thermal gelation, and both rely on the formation of disulfide bonds and hydrophobic groups [58]. It is generally believed that the formation of a protein gel network is caused by the interaction between proteins and solvents, as well as the attraction and repulsion between polypeptide chains, which is related to hydrogen bonds, hydrophobic interaction, electrostatic interaction and disulfide bond. For example, the high content of −SH groups and S-S groups is conducive to the formation of an intermolecular network structure, and the high content of hydrophobic groups tends to result in the formation of a firm network structure [67,68,69].

**Figure 2:**
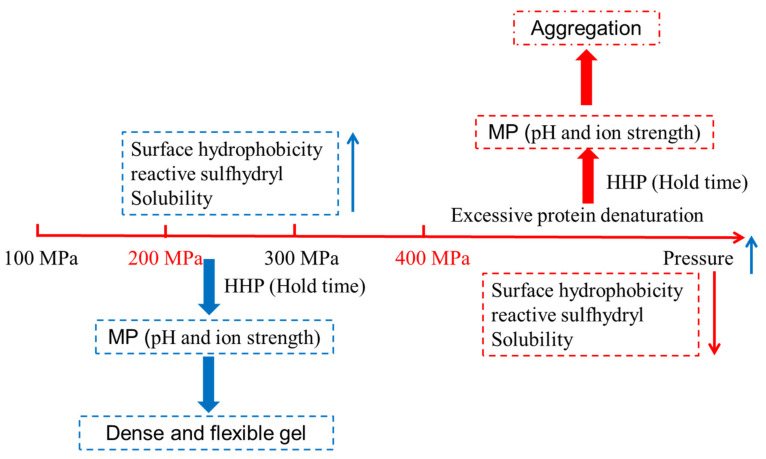
Schematic diagram of the mechanism of pressure-induced protein gel.

HHP treatment can lead to protein unfolding [25]. Figure 2 showed the mechanism of pressure-induced protein gel. The unfolding of protein is more likely to expose the reactive groups (especially hydrophobic groups), facilitating the enhancement of hydrophobic interactions, hydrogen bonding, and electrostatic interactions [26]. Therefore, proteins with a large molecular weight and a high hydrophobic amino acid content are easily able to form a stable three-dimensional network structure. HHP treatment can also expose the sulfhydryl groups inside the protein molecules, which is conducive to the formation or exchange of disulfide bonds [9]. The existence of a large number of hydrophobic groups and disulfide bonds can strengthen the intermolecular network and facilitate the formation of irreversible gel. Angsupanich et al. noted that, although the pressure-induced myosin denaturation generated very different gels compared to the thermal process, the gelation mechanisms were similar [64]. Meanwhile, protein gelation also depends on the difference between the denaturation rate and the aggregation rate of myosin in the presence of HHP or heat. If the denaturation rate is slow, the functional groups in the protein molecules can fully expand and interact with each other to form a complete and ordered gel network. However, when the aggregation rate is relatively fast, the formation of stable chemical bonds between the peptides is difficult, resulting in disordered and rough gel products [70]. 

## 2. Effect of HHP on Meat Quality 

HHP treatment of meat has no significant effect on its flavor compounds, amino acids, vitamins or other small molecular substances when compared with traditional thermal processing [11,71]. As a promising emerging technology, the natural color, aroma, taste and nutrient contents of foods are retained to the greatest extent by reducing the degree of food processing as far as possible with HHP. Furthermore, the quality of foods can be improved, and the storage period can be prolonged [6].

The use of HHP as a non-thermal sterilization technique, with minimal impact on sensory quality and nutritional value, has received wide approval, and a variety of meat products have benefited from the application of HHP to ensure food safety and the extension of shelf life [72,73]. The deactivation of vegetative microbial cells usually occurs in the pressure range of 200 to 600 MPa at room temperature or in freezing facilities, which is the process often used in commercial and industrial condition [74]. The ability of HHP to disrupt microbes in meat products has been well documented in past studies [5]. High pressure can destroy the cell membranes of microorganisms and thereby deactivate them [75,76]. Endogenous (matrix properties) and exogenous (processing conditions) factors largely determine the inactivation efficiency of spoilage and pathogenic bacteria in meat product manufacturing. Pressure, temperature and duration time are considered to be the most important process parameters [74].

Color is considered to be the most important factor influencing consumers to judge the freshness of food products. There are three main parameters for evaluating color, namely L* value (brightness), A* value (redness) and B* value (yellowness). Under the condition of 4 °C and 100–200 MPa for 15 min, the muscle brightness of flounder increased continuously, and the brightness of cod also increased continuously under the conditions of 220–300 MPa for 5 min [77]. The treatment of carp muscle at 300 MPa and 20 °C for 10 min reduced its transparency and increased its L * value; while it had a similar appearance to cooked carp muscle after treated being treated at 500 MPa and 20 °C for 10 min [78]. HHP treatment causes color changes in meat products, depending on the effect of stress on the denaturation of proteins (mainly myoglobin). Generally speaking, soft pressure treatment will increase the brightness of meat products and decrease the redness. The color change after pressure treatment differs from meat to meat. At present, the effect of the mechanism of HHP on the color change of meat products is not clear and may be related to the degeneration of myoglobin and myofibrillar protein and the oxidation of lipid and protein [13].

**Figure 3:**
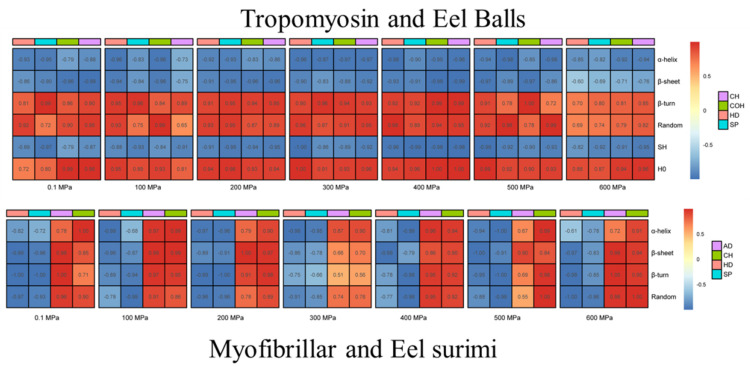
Correlation analysis of tropomyosin and Eel balls/myofibrillar protein and eel surimi (in our work).

Texture is one of the important indexes of meat products, among which hardness and elasticity are the most studied [79]. Hardness is considered to be the most important index for consumer recognition of fish [80]. It is well known that MPs are mainly responsible for the textural properties of processed meat products [81]. With increasing pressure, the hardness, springiness and chewiness values of red swamp crayfish (*Procambarus clarkia*) initially increased sharply, then decreased slightly, but all were higher than those of the control [82]. In our work, the texture parameters including hardness, springiness, chewiness and cohesiveness of eel balls were significantly improved with HHP treatment, and the optimum treating pressure was 400 MPa [9,36]. As for the eel surimi, the hardness, chewiness, gel strength and water-holding capacity increased significantly as well under HHP treatment, while the viscosity decreased [9,36]. The hardness, elasticity, mastic ability and cohesiveness increased by 3.00, 1.61, 5.96 and 1.23 times, respectively, when treated at 400 MPa for 15 min. In our work [9,36], further exploration of the protein structure revealed that the texture parameters of eel products were negatively correlated with the α-helix and β-sheet of proteins, but positively correlated with the random coil and β-turn of proteins (Figure 3). These results indicated that food textures can be directly related to the secondary and tertiary structures of MP.

Protein oxidation not only leads to the deterioration of food color and texture, but also to the loss of nutrients (e.g., essential amino acids) and a reduction in protein digestibility [83,84]. In a recent review [85], the mechanism of HHP on lipid oxidation and protein oxidation in meat products was examined in detail. Pressure seems to be critical for the initiation of lipid oxidation, which is probably related to effects on the non-heme iron in meat [85]. The oxidation of lipids and proteins can be closely related depending on the type of meat, applied treatment and the method used to evaluate the reaction [85,86]. As observed, the effects of HHP may be greater for chilled meat products than for cooked meat products [4,23]. Better control of the quality of the meat products subjected to HHP treatments requires strict control of the entire process, from raw materials to preservation and consumption.

HHP has been proven to be effective in improving the color and texture of meat products (Table 3). Generally, excessive salt consumption causes cardiovascular problems and hypertension [87]. Muscle-gelled foods normally contain 2–3% NaCl [87]. In recent investigations, HHP has been found to be an effective method to reduce the addition of salt [88]. Cando et al. found that with HHP pre-treatment prior to heating, the addition of salt could be reduced to 0.3% (NaCl) compared to 3% (NaCl) addition in the single thermal treatment technique, with both achieving similarly high quality surimi gel [87]. Orel et al. suggested that HHP developed sodium-reduced chicken breasts [89]. Yang et al. reported that HHP not only decreased the salt content, but also reduced the fat concentration in sausages with maintained textural qualities [90]. Furthermore, HHP is also considered to be a potential method for developing low phosphate meat products [88] HHP can increase digestive susceptibility with the exposure of cleaving sites and the acceleration of proteolysis during aging [91], in contrast with thermal treatment (excessive heating) which hinders the digestion of meat proteins [92,93]. 

## 3. Conclusions and Prospect 

Although HHP is considered to be an effective technology for improving the quality of meat as well as for the production of muscle-gelled foods, a certain threshold of treatment intensity is required for meat processing. However, the change in MP structure is revealed to be the main factor underlying the improvement in the quality of meat products. The spatial conformation of MP changes under HHP treatment, leading to changes in the product color and texture, and the mechanisms demonstrate the interdependence of the processes. However, meat is a complex system, containing not only MP but also other macromolecular proteins, polysaccharides and small molecules. Thus, the interactions between the different components under HHP treatment, together with their effects on the quality of meat products, require further in-depth research, particularly with regard to the molecular mechanism of the process. 

## Figures and Tables

**Table 1:** Changes in myofibrillar protein structure under HHP, including source, reaction conditions and relevant results.

Scheme	Protein	Treatment Conditions	Secondary Structure	H_0_ and −SH	Reference
Silver carp (*Hypophthalmichthys molitrix*)	Myofibrillar	200 to 500 MPa for 10 min at 20 °C	100 MPa, no significant changes; 200 to 300 MPa, slight changes; above 400 MPa, α-helix fraction gradually decreased.	≥300 MPa, the content of H_0_ and –SH increased.	[32]
Breast meats from Chicken	Myofibrillar	100 to 500 MPa for 10 min	With increasing pressure, α-helix and β-sheet transformed into random coil and β-turn.	With increasing pressure, the content of H_0_ and –SH increased.	[26]
Pork	Myofibrillar	200, 400, 600 and 800 MPa for 10 min at 5 or 20 °C	Solubility reduced because of aggregation.		[49]
Eel surimi	Myofibrillar	100–600 MPa for 15 min at 25 °C	100–600 MPa, α-helix converted into a random coil and β-turn.	The content of H_0_ increased, total –SH reduced and reactive –SH increased.	[9]
*Trichiurus Haumela* Surimi	Myofibrillar	300, 350, 400 and 450 MPa for 5 min at 18 ± 2 °C	The contents of α-helix and β-turn reduced, β-sheet and random coil increased.		[37]
Large white sow *biceps femoris*	Myofibrillar	200, 400 and 600 MPa for 6 min at 20 ± 4 °C	High pressure promoted the α-helix reduction and the increase in β-sheet structures.		[34]
Pre-rigor rabbit muscles	Myofibrillar	100, 200 and 300 MPa for 15 or 180 s at 25 °C		100 MPa, the content of H_0_ and reactive –SH slightly increased, above 200 MPa, the H_0_ reduced.	[50]
Hake	Myofibrillars	150, 250 and 500 MPa for 10 min	α-Helix reduced, β-sheet, β-turn and random coil increased.		[51]
Chicken breast muscle (Pectoralis major)	Freeze-dried myofibrillar	69, 103 and 138 MPa, high pressure homogenizer	Above 103 MPa, α-Helix and β-turn conversion to β-sheet structures occurred.		[52]
Threadfin bream (*Nemipterus spp.*)	Actomyosin	200, 400 and 600 MPa for 10, 30 and 50 min at room temperature		With pressure increasing, the H_0_ significantly increased, and the increasing speed of H_0_ slowed with treatment time incresing; the total –SH obviously reduced with pressure and time increasing, and the reactive –SH increased.	[14]
Snakehead	Actomyosin	100–600 MPa for 15 min at room temperature	100–600 MPa, α-helix converted into random coil and β-turn.	200–600 MPa, the H_0_ and reactive –SH increased.	Our work (Unpublished)
Hake (*Merluccius merluccius*)	Sarcoplasmic	200, 400 and 600 MPa for 6 min at 20 °C	α-Helix reduced, β-sheet, β-turn and random coil increased.	Above 200 MPa, the H_0_ and reactive –SH increased.	[53]
Eel surimi	Tropomyosin	100–600 MPa for 15 min at room temperature	200–400 MPa, β-sheet converted into random coil and β-turn; 500–600 MPa, α-helix converted into β-turn and β-sheet.	With increasing pressure, the content of H_0_ increased and –SH reduced.	[36]
Three-month-old male New Zealand rabbits	Myosin	100, 150 and 200 MPa for 2 min at 10 °C	α-Helix content reduced as the pressure increased (≤150 MPa), β-sheet, β-turn and random coil increased after HHP.		[54]
Eel surimi	Actin and Myosin	400 MPa for 15 min at room temperature	400 MPa, α-helix of actin significantly decreased, and β-sheet, β-turn and random coil increased; α-Helix and β-turn of myosin decreased, and random coil increased.		Our work (unpublished)
Red abalone (*Haliotis rufescens*)	Protein	200, 300, 400 and 500 MPa for 5 min	The intermolecular β-sheet structure was disrupted at 200 MPa; the 3_10_-helix structure significantly reduced at 300 MPa. β-turn was formed at 300, 400, and 500 MPa.		[28]
Palm ruff species	Protein	450 and 550 MPa for 3 and 4 min at ambient temperature	HHP reduced α-helix and increased β-sheet.		[55]

**Table 2:** Effects of HHP on myofibrillar gel.

Source	Protein	Treatment Conditions	Results	Reasons	References
Ice-bathed chicken breast meat	Myofibrillar	100, 150, 200, 250 and 300 MPa for 10 min Containing 0.3 M NaCl and 20 mM CaCl_2_	At 200 MPa (optimal pressure), the MP-Ca gel was strongest and the WHC was highest.	The solubilization of myosin heavy chain and actin increased, aggregation ability of MP reduced and the Tyr and Trp residues increased exposure (0.1 to 200 MPa).	[57]
Six-week-old commercial broilers	Myofibrillar	100, 200, 300, 400 and 500 MPa (±10 MPa) with a speed of 3.5 MPa/s for 10 min	At 200 MPa (optimal pressure), the MP gel was strongest.	The solubilization of MP increased below 200 MPa.	[26]
*M. psoas* from rabbits	Myosin	100–400 MPa at 20 °C for 10 min containing 0.6 M NaCl, pH 6.5	Gels were formed at 400 MPa.	Myosin was unfolded, resulting in exposure of hydrophobic groups, the heavy chain became very weak and associated to form gels.	[42]
Cod and turkey muscle	Myofibrillar/myosin	200–800 MPa for 20 min at ambient temperature	Pressure-induced myosin denaturation leads to very different gels to heat.	Disulfide bonds and hydrophobic interactions.	[58]
Bovine muscles (*biceps femoris*)	Myofibrillar	0–600 MPa for 0–1800 s	HHP can change the gel properties.	Surface hydrophobicity and reactive sulfhydryl groups increase around 450 MPa, which induced significant decreases in viscosity and solubility.	[59]
Live round tilapia (*Orechromis niloticus*)		50–300 MPa for 30 min	Above 200 MPa, Gels were softer, more viscous.	Hydrogen bonds and hydrophobic interactions.	[60]
Chicken breast meat	Myofibrillar	100, 200, 300, 400, 500 MPa (±10 MPa) for 10 min	200 MPa was the optimum pressure for the WHC of MP gel.	HHP caused more disulfide bond and stronger electrostatic repulsion, and hydrophobic interactions.	[44]
Tilapia (*Oreochromis niloticus*) surimi		0, 100, 200, 300, and 400 MPa for 15 min	Gels formed by pressurization were dense and flexible.	Disulfide bonds played a significant role in gel formation, and tyrosine residues involved in hydrogen bond formation with a lower intensity ratio.	[61]
Golden threadfin bream (*Nemipterus virgatus*)	Myosin contained deacetylated konjac glucomannan	100, 200, 300, 400 and 500 MPa (±15 MPa) for 5 min	Suitable pressure level (0.1–300 MPa) could improve thermal gelling ability and gelation properties.	Hydrophobic interactions were dominating mechanism to enhance gel strength.	[62]
Greater lizardfish (S. tumbil) surimi		Heating treatment (control), optimal high-pressure treatment (P), optimal MTGase treatment (M), MTGase combined with high pressure (MP), MTGase combined with setting, and high pressure (MSP)	MSP exhibited the highest gel strength, a higher water-holding capacity than the control sample.	Greater proportion of hydrogen bonds formed and non-disulfide covalent bonding catalyzed by MTGase under HHP.	[63]
Chicken breast meat (*Musculus pectoralis major*)	Myofibillar	Pressure/temperature:0.1, 200 or 400 MPa at 20 or 75 °C for 30 min	The gel induced by heating under pressure had a uniform porous microstructure and higher water-holding capacity.	High pressure in the HUP treatment prevented (HHP combined simultaneously with heating) myofibillar from being heat-denatured and reduced protein secondary structural transformation and hydrophobic residues exposure.	[64]

**Table 3:** Effect of HHP on meat quality.

Source	Treatment Conditions	Results	Optimal Conditions	References
Frozen chicken breast meat	100, 200 and 300 MPa for 10 min Compression fluid: water	Below 200 MPa the water-holding capacity of meat increased.		[94]
Frozen chicken breast	100, 200, 300, 400, 500 and 600 MPa for 1 min or/and 3, 5, 7 and 9 min Compression fluid: propylene glycol and distilled water (30:70 *v/v*).	The parameters of color and texture have significantly enhanced under HHP.	500 MPa for 1 min	[95]
White chicken meat	50, 100, 200 or 300 MPa for 60, 120 or 180 s	Lower NaCl concentrations along with HHP treatment improved the color and texture of white chicken meat.	Color: 300 MPa and 1.5% NaClCooking loss: 100 MPa and 1.5% NaCl	[96]
Mackerel fillets	100, 300 and 500 MPa for 2 and 5 min	Lightness (L*) increased and redness (a*) decreased at 300 and 500 MPa. Hardness and springiness increased at 500 MPa.	Microbial inactivation: 500 MPa for 2 min and 300 MPa or 500 MPa for 5 min	[97]
Black tiger shrimp (*Penaeus monodon*)	100, 270 and 435 MPa for 5 min at room temperature	HHP increased the hardness, which showed a reduction trend during storage. Shelf life was extended to 15 days at 435 MPa, compared with 5 days in control sample.	435 MPa for 5 min	[98]
Eel *(Anguilla japonica)* Surimi	100, 200, 300, 400, 500 and 600 MPa for 15 min at 20 °C Compression fluid: water	Hardness, adhesiveness, and chewiness of surimi had significantly increased compared to control above 400 MPa.The hardness, adhesiveness, chewiness, and springiness of surimi showed a negative relationship with the α-helix and β-sheet, and a positive correlation with random coil and β-turn.	400 MPa for 15 min	[9]
Marinating Tan mutton	100, 200, 300 and 400 MPa for 15 min at 25 ± 1 °C Compression fluid: water	200 MPa can effectively enhance the tenderness of lamb leg meat. The lightness value increased significantly with the increasment of pressure	200 MPa for 15 min	[99]
Bovine (*M. longissimus dorsi, LD*) steaks	175 and 600 MPa for 3 and 10 min at room temperature	The visual appearance and texture of the meat have obviously changed at 600 MPa.	600 MPa	[100]
Frozen rabbit muscles	100, 200 and 300 MPa for 3, 9 and 15 min at 25 °C Compression fluid: water	200 MPa for 3 min was the threshold for effective HHP treatment under the used conditions.Above 200 MPa for 3 min helped to produce high yielding and juicy gel-type products.	Above 200 MPa	[101]
Eel surimi	100, 200, 300, 400,500 and 600 MPa for 15 min at 25 °C Compression fluid: water	The hardness, springiness, chewiness and cohesiveness have significantly increased above 400 MPa.The texture profiles of eel balls showed a negative relationship with the α-helix, β-sheet and SH content.	400 MPa	[36]
Berian dry-cured “salchichón” (DCS) and dry-cured loin (DCL)	600 MPa for 8 min and stored at 4 and 18 °C	The *Listeria monocytogenes* could be significantly ruduced under 600MPa for 8 min, the reductive speed is faster when storage temperature is at 18 °C.	600 MPa for 8 min and stored at 18 °C	[102]
Cooked fish batters from fresh tilapia (*Oreochromis niloticus*) fillets	UV-C treatment (UV25%, UV + HHP25%, UV50%, and UV + HHP50%) and HHP treatment (HHP25%, UV + HHP25%, HHP50%, and UV + HHP50%) HHP: 300 MPa for 5 min at 25 °C	The UV-C and HHP (signal treatment) could effectively retained the cooking loss, color and texture parameters, and salty taste reduced by 25%, whereas combined UV-C and HHP were not beneficial to its cooking loss and salty taste.	UV-C at 0.310 J/cm^2^ or HHP at 300 MPa for 5 min at 25 °C	[103]
Frozen skinless and boneless chicken breasts	300 MPa for 5 min after tumbling (HHP-T1); 600 MPa for 3 min to the final product (HHP-T2)	HHP-T1 enhanced cooking loss of the ready-to-eat chicken breasts, hardness and sensory; HHP-T2 could extend the shelf life.		[97]
Beef jerky	0.1 MPa (HHP-0.1), 100 MPa (HHP-100), 200 MPa (HHP-200), or 300 MPa (HHP-300); combined with HHP and moisture regulators at a pressure of 0.1 MPa (MR + HHP-0.1), 100 MPa (MR + HHP-100), 200 MPa (MR + HHP-200), or 300 MPa (MR + HHP-300)	The MR + HHP-200 samples showed the similarity of the tenderness to samples treated only with HHP-300; the addition of MR increased the water content of beef jerky.	MR + HHP-200	[104]

## Data Availability

Not applicable.
